# Radioiodine ablation in a haemodialysis-dependent patient with papillary thyroid carcinoma

**DOI:** 10.1530/EDM-25-0153

**Published:** 2026-04-01

**Authors:** Aisha Salem Alzahmi, Thoraia Awad Abdelrahman, Asif Moin, Hiba Drwesh, Mohammed Hassan Al Ali, Hessa Ebrahim Boharoon

**Affiliations:** ^1^ Department of Internal Medicine, Sheikh Khalifa Medical City, Abu Dhabi, UAE; ^2^ Department of Nuclear Medicine, Sheikh Khalifa Medical City, Abu Dhabi, UAE; ^3^ Department of Radiology, Sheikh Khalifa Medical City, Abu Dhabi, UAE; ^4^ Department of Endocrinology, Sheikh Khalifa Medical City, Abu Dhabi, UAE; ^5^ Department of Otolaryngology–Head and Neck Surgery, Sheikh Khalifa Medical City, Abu Dhabi, UAE

**Keywords:** case report, papillary thyroid carcinoma, radioiodine ablation, end-stage renal disease, hemodialysis

## Abstract

**Summary:**

The management of differentiated thyroid cancer with radioactive iodine (RAI) in patients with end-stage renal disease (ESRD) on haemodialysis is particularly challenging because of impaired iodine clearance, prolonged radiation exposure and safety concerns for healthcare personnel. We present the case of a 54-year-old male on thrice-weekly maintenance haemodialysis who was incidentally diagnosed with papillary thyroid carcinoma during pre-transplant assessment. He underwent total thyroidectomy followed by adjuvant RAI ablation, planned on the basis of the available literature. A reduced dose of 50 mCi (1.85 GBq) was administered after stimulation with recombinant human TSH, and dialysis was scheduled at 48 h post-therapy to optimize uptake, with additional sessions on Days +3 and +6. Ablation was successful, with iodine-avid tissue seen on post-therapy imaging and no significant complications observed. This case demonstrates that with individualized dosing, tailored dialysis scheduling and multidisciplinary coordination, RAI ablation can be performed safely and effectively in patients with ESRD on haemodialysis, despite the absence of standardized guidelines.

**Learning points:**

- A reduced radioactive iodine (RAI) dose can achieve successful ablation in dialysis-dependent patients, minimizing systemic radiation exposure and marrow toxicity without compromising efficacy.
- The timing of the first haemodialysis session after RAI administration is critical to ensure effective thyroidal ablation while minimizing radiation toxicity.
- There are no standardized guidelines for RAI ablation in end-stage renal disease (ESRD) patients on haemodialysis, necessitating case-by-case multidisciplinary planning.

## Background

Thyroid cancer is the most common endocrine malignancy, and its management often involves a combination of surgery, radioactive iodine (RAI) therapy and thyroid hormone suppression. However, in patients with end-stage renal disease (ESRD) requiring haemodialysis, RAI therapy poses unique challenges due to altered iodine metabolism and the need to mitigate radiation exposure to dialysis staff and equipment. Notably, there are no specific guidelines for administering RAI therapy for thyroid cancer in patients with ESRD undergoing haemodialysis, creating a gap in evidence-based management strategies. This case highlights the complexities of managing a patient with ESRD on haemodialysis who was awaiting kidney transplantation and was diagnosed with papillary thyroid carcinoma (PTC) requiring RAI therapy.

## Case presentation

A 54-year-old male with a medical history significant for type 2 diabetes mellitus, hypertension and ESRD on dialysis awaiting kidney transplantation presented to the thyroid cancer clinic following an incidental finding of a thyroid nodule during pre-transplant assessment.

## Investigations

Thyroid ultrasound demonstrated a well-circumscribed, isoechoic right-lobe nodule measuring 3.4 × 3.4 × 5.2 cm, classified as thyroid imaging reporting and data system (TI-RADS) 3, along with two spongiform nodules in the right and left lobes, both classified as TI-RADS 1. Ultrasound-guided fine-needle aspiration (FNA) of the large right thyroid nodule was performed, and the result was consistent with malignant PTC ‘Bethesda System Category VI’.

## Treatment

A multidisciplinary team meeting involving an endocrinologist, nuclear medicine physician, nephrologist and clinical pharmacist was convened, and the decision was made to proceed with total thyroidectomy followed by RAI ablation to achieve complete disease eradication prior to kidney transplantation and subsequent immunosuppression.

The patient subsequently underwent total thyroidectomy with central neck dissection. Postoperatively, his recovery was uneventful, and he was discharged on levothyroxine 125 μg daily. Histopathological examination revealed a unifocal PTC, follicular variant, located in the right lobe, measuring 5 cm in the greatest dimension. The surgical margins were free of tumour, and the final staging was pT3aN0Mx. Based on the 2015 American Thyroid Association (ATA) guidelines, the patient was categorized as intermediate risk. Given his planned future kidney transplantation, and in order to minimize the risk of recurrence, adjuvant radioiodine therapy was considered appropriate.

In view of the importance of timing in dialysis-dependent patients, the day of radioactive iodine (RAI) administration was designated as Day 0, and subsequent events are described relative to this reference point. On Day −2, the patient received a single intramuscular injection of recombinant human TSH (rhTSH: 0.9 mg). The second dose was withheld because the TSH level measured on Day −1 had already exceeded 200 mIU/L, and further stimulation was considered unnecessary in the setting of impaired renal clearance.

Prior to RAI administration, all personnel involved in the patient’s care, including dialysis nursing staff, received dedicated training on radiation safety, proper handling procedures and exposure minimization strategies.

On the morning of Day 0 (the day of RAI administration), the patient underwent routine haemodialysis. He was subsequently admitted to the dedicated radioiodine isolation room, where 50 mCi (1,850 MBq) of I-131 was administered.

Before patient admission, our dedicated radioiodine isolation room had been modified to enable in-room haemodialysis. The dialysis machine was positioned inside the room in advance, and mobile lead shields were arranged between the patient’s bed and the dialysis machine, leaving a narrow access window. This configuration allowed the patient to extend only the forearm through the opening for arteriovenous (AV) fistula cannulation, thereby minimizing radiation exposure to dialysis personnel.

Following RAI administration, haemodialysis sessions were performed within the isolation room on Day +2, Day +3 and Day +6. Each dialysis session was conducted by a single dialysis nurse; they were asked to spend less time with the patient in keeping with as low as reasonably achievable (ALARA) principles. Continuous vital signs were displayed on an external monitor to facilitate remote observation, and the patient was provided with a call buzzer to request assistance as needed.

**Figure 1:**
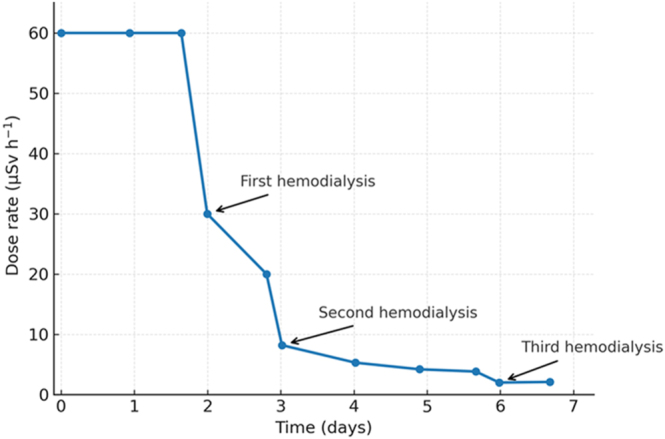
Time course of 1-m dose rate (μSv·h^−1^) after therapeutic 131-iodine (1,850 MBq) in our haemodialysis-dependent patient.

For radiation monitoring, dialysis staff wore personal dosimeters beneath standard personal protective equipment to document cumulative exposure during each session. Nuclear medicine personnel measured whole-body radioactivity at predefined intervals and after each haemodialysis session at a distance of 1 m from the patient; results are summarized in Fig. 1. Post-procedure contamination surveys were performed on the dialysis equipment and surrounding areas. All disposable materials and dialysate effluent were treated as radioactive waste and stored for decay prior to disposal.

In addition to staff-protection measures, several strategies were implemented to minimize radiation exposure to the patient, particularly given the anticipated delayed clearance of I-131 in the end-stage kidney disease. The patient fasted for 2 h prior to RAI administration to reduce gastric mucosal radiation exposure. Following ablation, he was instructed to shower two to three times daily to facilitate removal of radioactive sweat and reduce cutaneous contamination.

To mitigate salivary gland radiation exposure, sugar-free sour lollies were initiated 24 h after RAI administration to stimulate salivary flow and promote iodine clearance from the glands. These measures were continued throughout the isolation period alongside scheduled haemodialysis sessions.

**Figure 2:**
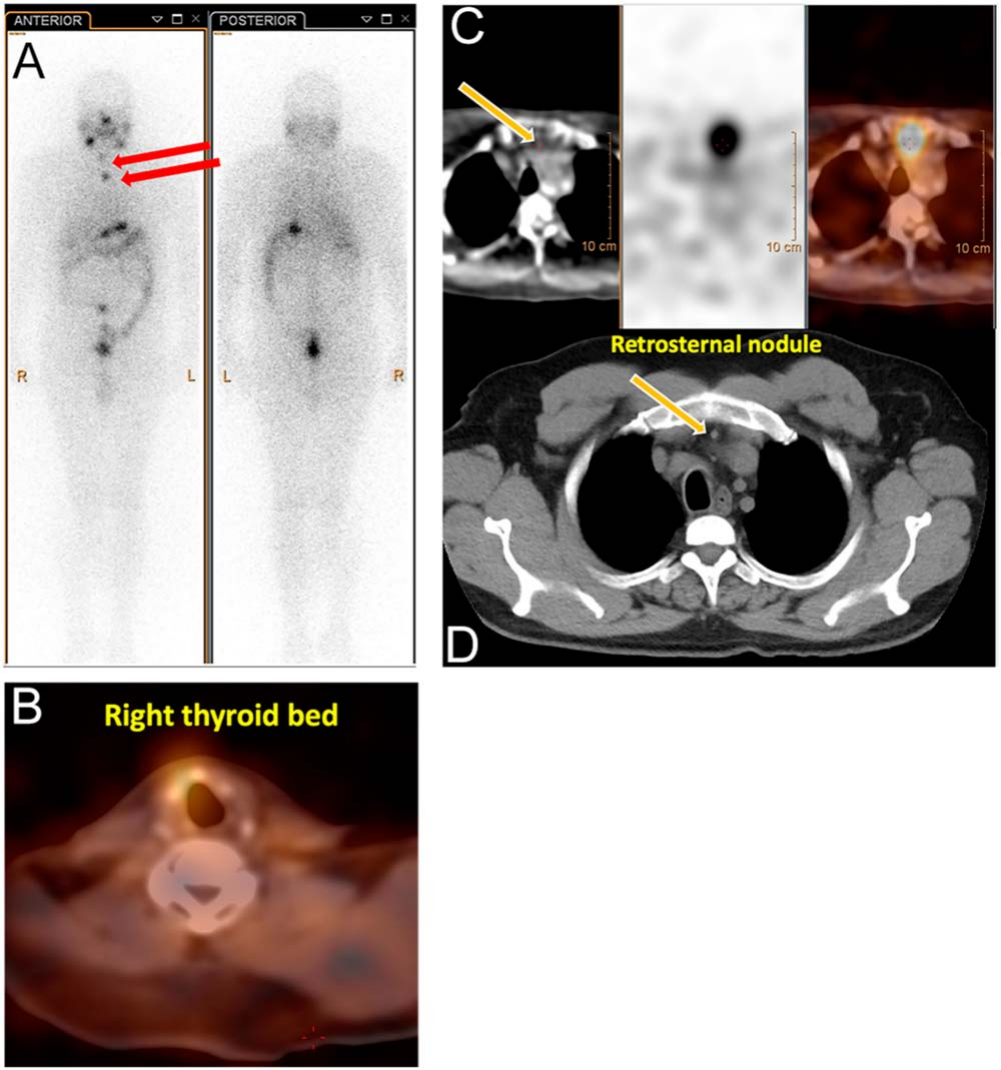
A focal area of increased radiotracer uptake is noted in the right thyroid bed and retrosternal region on whole-body images (A). These findings are more precisely localized on SPECT/CT images (B and C). Corresponding diagnostic CT performed on the same day (D) demonstrates a well-defined soft tissue nodule in the retrosternal area with superior anatomical resolution %.

During the admission, a whole-body I-131 scan (WBS) was performed, which demonstrated iodine-avid residual tissue in the right anterior thyroid bed and an iodine-avid focus in the retro-manubrial area. Due to concerns for possible bone metastases, a CT chest with contrast was performed, which showed no focal lytic or sclerotic lesions in the sternum (Fig. 2).

## Outcome and follow-up

The patient met radiation safety criteria for discharge on Day +3, in accordance with local regulations permitting discharge once the exposure rate at 1 metre is ≤ 30 μSv/h. However, discharge was postponed until Day +8 due to delayed cross-sectional imaging and social circumstances. He was subsequently discharged on suppressive levothyroxine therapy with outpatient follow-up arranged, targeting a TSH of 0.1–0.5 IU/mL. Following the final haemodialysis session, a contamination survey of the portable dialysis machine revealed no detectable radioactivity; the machine was maintained in isolation for two weeks before being returned to routine clinical service.

The patient continued regular follow-up in the endocrinology clinic, demonstrating excellent clinical and biochemical response with undetectable thyroglobulin levels and negative anti-thyroglobulin antibodies, and he remained clinically stable on suppressive levothyroxine therapy and proceeded with his kidney transplant preparation.

## Discussion

PTC is the most common subtype of differentiated thyroid cancer (DTC) and is typically managed with total thyroidectomy followed, in selected cases, by adjuvant radioiodine (I-131) ablation according to risk stratification. The goal of I-131 therapy is to deliver an effective radiation dose to residual thyroid tissue or metastatic disease while minimizing haematologic and systemic toxicities. Since I-131 is predominantly eliminated via the kidneys, patients with advanced renal impairment experience delayed clearance, prolonged radiation exposure and increased risk of toxicity (1). These pharmacokinetic changes necessitate individualized treatment strategies, including activity modification, carefully coordinated dialysis scheduling and tailored radiation safety protocols for both healthcare personnel and waste management.

In our case, given the anticipated need for kidney transplantation and the requirement for subsequent immunosuppression, the multidisciplinary team (MDT) elected to proceed with radioiodine ablation to optimize oncologic control prior to transplantation. Recognizing the unique challenges posed by performing ablation in a haemodialysis (HD) setting, we reviewed the available literature to guide treatment planning and ensure both efficacy and safety.

In patients with ESRD on dialysis, both thyroid hormone withdrawal (THW) and recombinant human TSH (rhTSH) have been applied successfully to achieve the TSH elevation required for effective radioiodine uptake. To date, no randomized controlled trials have compared these two strategies in dialysis-dependent individuals, and evidence is limited to case reports and small series. We elected to use rhTSH in our patient, as levothyroxine withdrawal is consistently associated with symptomatic hypothyroidism, leading to impaired quality of life and reduced functional capacity. Moreover, studies have shown that even in patients with normal renal function, THW can transiently lower glomerular filtration rate and contribute to electrolyte disturbances such as hyponatremia and hyperkalaemia (2, 3), whereas rhTSH avoids these metabolic effects. An important consideration in dialysis is the markedly slower clearance of rhTSH, which has prompted dose modification. Recently, several reports have described successful use of a single 0.9 mg rhTSH injection administered 48 h prior to radioiodine therapy (4, 5, 6), consistently achieving target TSH while reducing costs, logistical complexity and the risk of prolonged supratherapeutic levels. In line with these reports, we adopted the single-dose approach in our patient, whose repeat TSH measurement prior to ablation already exceeded 200 mIU/L.

There are conflicting recommendations in the literature regarding radioiodine dose. Some authors suggest lowering the dose to limit the whole-body and marrow exposure (4, 5, 6), others kept the usual dose (7), and some even recommended to increase the dose (8). For our patient, we selected a reduced-dose regimen for several reasons. He did not have aggressive metastatic disease, which provided some flexibility for dose adjustment. In addition, as discussed previously, he had no significant residual renal function (dialysis-dependent), which strongly favoured a conservative approach to minimize the risk of marrow suppression and systemic toxicity. Furthermore, our patient was classified as intermediate risk according to the ATA guidelines, and in line with other published case reports of intermediate-risk patients, the use of a reduced dose of 50 mCi (1,850 MBq) achieved acceptable ablation and disease control (1, 9).

The scheduling of haemodialysis relative to RAI administration is critical; if dialysis is done too early, significant radioiodine may be removed before uptake by thyroid remnants; if too late, prolonged retention increases the systemic exposure. In our case, haemodialysis was performed on Day 0 (prior to RAI administration) and subsequently on Day +2, Day +3 and Day +6. Our decision to make the first dialysis session to 48 h was based on the intention to allow more complete thyroidal uptake, particularly given the reduced administered dose. This approach is supported by a published case report that showed patients on haemodialysis demonstrate only ∼6% uptake of I-131 if first dialysis post I-131 dose was at 24 h and ∼10% at 48 h, suggesting that dialysis before 48 h may risk undertreatment (9). Although some case reports initiated dialysis within 24 h, they are often in the context of unchanged or increased dose of I-131.

## Conclusion

This case illustrates that radioiodine ablation can be delivered safely and effectively in a haemodialysis-dependent patient. In our case, the use of recombinant human TSH, a reduced dose of 50 mCi (1.85 GBq) and scheduling the first dialysis at 48 h allowed effective ablation while maintaining radiation safety for both the patient and healthcare staff. This case emphasizes the importance of multidisciplinary collaboration and highlights the need for further evidence-based guidelines in this complex clinical setting.

## Declaration of interest

The authors declare that there is no conflict of interest that could be perceived as prejudicing the impartiality of the work reported.

## Funding

This work did not receive any specific grant from any funding agency in the public, commercial or not-for-profit sector.

## Patient consent

Written informed consent for publication of their clinical details and clinical images was obtained from the patient.

## Author contribution statement

ASA collected the clinical data and drafted the initial manuscript. TAA contributed to radioiodine preparation, administration and radiation safety procedures. AM assisted in imaging acquisition, radioiodine dosimetry and technical aspects. HD contributed to patient care, data collection and coordination during hospitalization and follow-up. MHAlA performed the thyroidectomy and contributed to surgical expertise and input to the manuscript. HEB provided endocrinology expertise, supervised overall management, critically reviewed and revised the manuscript and approved the final version. The named authors meet the International committee of Medical Journal Editors (ICMJE) criteria for authorship of this manuscript, take responsibility for the integrity of the work as a whole and have given final approval for this version to be published.
